# Transdiagnostic monoamine-based subtyping for attention-deficit/hyperactivity disorder and autism spectrum disorder via unsupervised machine learning

**DOI:** 10.1007/s00702-026-03206-z

**Published:** 2026-07-27

**Authors:** Masatoshi Yamashita, Qiulu Shou, Sayo Hamatani, Hideo Matsuzaki, Masanori Fujieda, Yoshiyuki Hirano, Kuriko Kagitani-Shimono, Hidehiko Okazawa, Yoshifumi Mizuno

**Affiliations:** 1 Research Center for Child Mental Development, University of Fukui, Fukui, Japan; 2 Research Center for Child Mental Development, Hamamatsu University School of Medicine, Shizuoka, Japan; 3 United Graduate School of Child Development, The University of Osaka, Kanazawa University, Hamamatsu University School of Medicine, Chiba University, University of Fukui, Osaka, Japan; 4 Department of Child and Adolescent Psychological Medicine, University of Fukui Hospital, Fukui, Japan; 5 Research Center for Child Mental Development, Chiba University, Chiba, Japan; 6 Department of Pediatrics, Osaka University Graduate School of Medicine, Osaka, Japan; 7 Biomedical Imaging Research Center, University of Fukui, Fukui, Japan

**Keywords:** Brain structure, Cross-disorder phenotypic overlap, Monoamine metabolite, Neurodevelopmental disorder, Unsupervised machine learning

## Abstract

Neuroimaging and molecular studies have examined the etiology of attention-deficit/hyperactivity disorder (ADHD) and autism spectrum disorder (ASD). However, their findings remain inconsistent because of within-disorder heterogeneity and cross-disorder phenotypic overlap. We sought to identify monoamine-based subtypes across ADHD and ASD and clarify their brain structural characteristics. In 83 children with ADHD and/or ASD, we applied unsupervised machine learning (NbClust with K-means) to identify neurodevelopmental disorder (NDD) phenotypes using urinary monoamine metabolite (MM) profiles. Behavioral symptoms, cognitive performance, cortical surface area, and gray matter volume (GMV) were evaluated for each NDD phenotype and for 83 typically developing (TD) children as controls. Clustering identified two urinary MM-defined NDD phenotypes: NDD-A (*n* = 18, including 5 ADHD, 2 ASD, and 11 ADHD + ASD cases), characterized by high levels of 4-hydroxy-3-methoxyphenylglycol, 5-hydroxyindoleacetic acid, and homovanillic acid, and NDD-B (*n* = 65, including 16 ADHD, 19 ASD, and 30 ADHD + ASD cases), characterized by low levels of these metabolites. Urinary 4-hydroxy-3-methoxyphenylglycol levels correlated positively with social communication difficulties in NDD-A. NDD-B showed significantly lower cognitive control, cognitive flexibility, and inhibitory control than TD. Structurally, compared with TD, NDD-A showed significant surface area enlargement in the isthmus cingulate gyrus, whereas NDD-B exhibited significant GMV reductions primarily in fronto–opercular/orbitofrontal regions, with additional reductions in the superior parietal lobule and supramarginal gyrus. These findings suggest that urinary MM-defined NDD-A and NDD-B phenotypes are associated with distinct cognitive and brain structural characteristics. Such phenotype specificity may provide a novel framework for understanding within-disorder heterogeneity and cross-disorder phenotypic overlaps.

**Supplementary Information:**

The online version contains supplementary material available at https://doi.org/10.1007/s00702-026-03206-z.

## Introduction

Attention-deficit/hyperactivity disorder (ADHD) and autism spectrum disorder (ASD) are common neurodevelopmental disorders (NDDs) in children and adolescents, with prevalence rates of > 5% and > 1.5% (Baio et al. 2018; Thomas et al. 2015), respectively. ADHD symptoms include inattention, hyperactivity, and impulsivity, whereas ASD is characterized by difficulties in social communication and interaction, restricted and repetitive behavioral patterns, and atypical sensory responses (Lane et al. 2010). Although the Diagnostic and Statistical Manual of Mental Disorders (DSM-5) provides diagnostic criteria for these conditions, the symptoms of children with ADHD and ASD may not fit within the boundaries of a single disorder, as considerable clinical overlap (Grzadzinski et al. 2016), a high comorbidity rate (Simonoff et al. 2008), and within-disorder heterogeneity (Yamashita et al. 2024) have been observed. Additionally, ADHD and ASD are associated with executive function differences (Townes et al. 2023), increased likelihood of various psychiatric disorders, fatigue, and sleep disorder (Antshel et al. 2016; Axelsson et al. 2024), highlighting the neurobiological overlap. As ADHD and ASD may be viewed as different manifestations of the same overarching disorder (Antshel and Russo 2019), establishing NDD phenotypes based on biomarkers, such as magnetic resonance imaging (MRI) parameters and biochemical indices, is important for biologically informed classifications.

Previous MRI studies have reported that, compared with typically developing (TD) individuals, individuals with ADHD show decreased gray matter volumes (GMVs) in the frontal regions, precuneus, and basal ganglia (Moreno-Alcázar et al. 2016; Vilgis et al. 2016; Zhao et al. 2020). GMV reductions in the frontal-temporal regions, precuneus, amygdala, and cerebellum have also been reported in individuals with ASD compared with TD individuals (Li et al. 2019; Sato et al. 2017; Yang et al. 2018). Nonetheless, such reductions have not been reported in other studies (Bonilha et al. 2008; Seidman et al. 2011; Semrud-Clikeman et al. 2014). These inconsistent results may reflect the phenotypic heterogeneity of NDDs. Additionally, several studies have reported that ADHD and ASD share overlapping brain structural characteristics in regions such as the medial temporal lobe, inferior parietal cortex, and cerebellum (Brieber et al. 2007; Dougherty et al. 2016). However, these studies on brain structure have also not always yielded consistent results. Hence, while clinical guidelines exist for ADHD pharmacotherapy, clear biomarker-based criteria to guide individualized medication selection, particularly for individuals with co-occurring ADHD and ASD, remain limited, and definitive biomarkers for diagnosis have yet to be established.

Monoamines represent a candidate biomarker, given its association with the pharmacological targets of therapeutic drugs for ADHD and ASD, such as methylphenidate, atomoxetine, and risperidone (Hellings 2023; Mechler et al. 2022; Ota et al. 2021). Previous brain-based positron emission tomography (PET) studies have suggested alterations in dopaminergic (e.g., dopamine synaptic markers), noradrenergic (e.g., norepinephrine transporter binding), and serotonergic (e.g., serotonin transporter binding) systems in individuals with ADHD compared with control groups (Kautzky et al. 2020; Sigurdardottir et al. 2016; Volkow et al. 2009). In addition, human postmortem molecular and brain-based PET studies have reported dopaminergic (e.g., dopamine-related gene expression) and serotonergic (e.g., serotonin synthesis capacity and serotonin receptor density) alterations in individuals with ASD compared with control groups (Brandenburg et al. 2020; Chugani et al. 1999; Goldberg et al. 2009). Although these findings suggest that complex changes between monoamines are involved in the molecular mechanisms underlying these NDDs, the evidence has been inconsistent (Alabdali et al. 2014; Karlsson et al. 2013; Schalbroeck et al. 2021; Ulke et al. 2019; Wiers et al. 2018). This may be due to the diversity within these disorders as well as their neurobiological overlap, as ADHD and ASD are not single conditions, but rather represent an aggregate of heterogeneous mechanisms. In this context, investigating peripheral monoaminergic indices may help capture biologically relevant inter-individual variability. Additionally, ADHD and ASD have been associated with alterations in the peripheral monoaminergic system, as indicated by changes in the urinary excretion of monoamines and their metabolites, such as 4-hydroxy-3-methoxyphenylglycol (MHPG, a noradrenaline metabolite), 5-hydroxyindoleacetic acid (5-HIAA, a serotonin metabolite), and homovanillic acid (HVA, a dopamine metabolite) (Dvoráková et al. 2007; Gevi et al. 2016, 2020). As urinary monoamine metabolites (MMs) are transported from the brain via the organic anion transporter 3 system in the blood–brain barrier (Mori et al. 2003; Ohtsuki et al. 2003), they may serve as valuable noninvasive biomarkers of monoamine activity in the brain (Amin et al. 1992; Yamashita and Yamamoto 2021). Elucidating the brain structural characteristics associated with urinary MM subtypes of NDD may help to address the inconsistent findings associated with within-disorder heterogeneity and cross-disorder phenotypic overlap. Nevertheless, investigations of the brain structure of NDD subtypes to date have been limited to clinical symptom- and executive function-related subtypes (Cordova et al. 2020; Zhang et al. 2022), and little is known about NDD subtypes classified by urinary MM patterns.

We theorized that a better understanding of within-disorder heterogeneity and cross-disorder overlap may be helpful when considering prognosis or informing treatment planning. Thus, this study aimed to clarify this gap in knowledge by investigating whether transdiagnostic ADHD and ASD subtypes, defined by urinary MM profiles, would show differences in brain structure. We hypothesized that, when using an unsupervised machine learning approach, children with these NDDs would be categorized into at least two clusters based on urinary levels of MHPG, 5-HIAA, and HVA. We further hypothesized that these monoamine-based subtypes would show distinguishable brain structural profiles (e.g., regional GMV and cortical surface area) as compared with TD children, and that these structural profiles would differ between NDD subtypes. We also examined cognitive performance to support the interpretation and characterization of the identified subtypes.

## Methods

### Participants

The Research Ethics Committee of the University of Fukui approved the study protocol (Assurance No. FU-20220061) and the study procedures complied with the principles of the Declaration of Helsinki. All parents provided written informed consent, and all children provided assent for participation. The study involved 172 children (TD group: *n* = 86; NDD group: *n* = 86; age: 6 − 17 years). Children who were diagnosed with ADHD, ASD, or co-occurring diagnoses based on the DSM-5 were recruited through a hospital at the University of Fukui, Japan. TD children who did not receive special support education were recruited from the local community. All participants had a Wechsler full-scale intelligence quotient score ≥ 70 (Table 1); no history of neurological, cardiovascular, or psychiatric illness, no contraindication for MRI, and had discontinued medication under physician supervision based on half-life of each drug prior to the experiment. Data from three TD children were excluded from the analysis because of claustrophobia during the MRI scan. Furthermore, data from three children with NDDs were excluded because of physical deconditioning and claustrophobia during the MRI scan. Thus, data from 83 TD children and 83 children with NDDs were used in the final analysis (Table 1).

**Table 1:** Demographics of the typical development and neurodevelopmental disorder groups

Characteristic	TD (*n* = 83)	NDD (*n* = 83)	*P*-value
Age (years)	10.76 (2.97)	11.75 (3.13)	0.038
Education (years)	6.34 (2.92)	7.28 (3.06)	0.044
Sex (n)			
Male	39	74	< 0.001
Female	44	9	
Annual household income (JPY) (n)			
< 3 000 000	6	10	0.003
3 000 000–5 000 000	9	24	
5 000 000–7 000 000	20	17	
≥ 7 000 000	48	32	
Diagnosis (n)			
ADHD		21	NA
ASD	NA	21	
ADHD + ASD		41	
Handedness (n)			
Right	78	75	0.133
Left	3	8	
Ambidextrous	2	0	
Intelligence quotient (score)	106.54 (12.74)	96.67 (12.75)	< 0.001
Urine collection time period (n)			
Moring	31	29	0.948
Midday	30	31	
Afternoon	22	23	
Assessment season (n)			
Spring	24	17	0.020
Summer	28	22	
Autumn	11	28	
Winter	20	16	

Parameters are indicated as the mean (*SD*) or n. *P*-values for age, education, income, and intelligence quotient are based on *t*-tests for the comparison of the TD group with NDD group. *P*-values for sex ratio, urine collection time period distribution, and assessment season distribution are based on Chi-square tests for the comparison of the TD group with NDD group. *P*-value for handedness is from Fisher’s exact test for the comparison of the TD group with NDD group

*ADHD* attention-deficit/hyperactivity disorder, *ASD* autism spectrum disorder, *NA* not applicable, *NDD* neurodevelopmental disorder, *SD* standard deviation, *TD* typical development

### Psychological and cognitive measurements

The psychological questionnaire targeted the Japanese version of the Social Responsiveness Scale (SRS-2), Short Sensory Profile, Conners 3 Scale, Chalder Fatigue Scale, and Munich Chronotype Questionnaire. Furthermore, cognitive tests included the stop signal and spatial working memory tasks from the Cambridge Neuropsychological Tasks Automated Battery and parts A and B of the Trail-Making Test (TMT). For the details of each questionnaire and cognitive function task, see Online Resource 1.

### Determination of MMs

Prior to testing, the research team instructed the children and their parents or guardians to ensure that the children refrained from intense physical activity and from consuming caffeine-containing beverages (e.g., coffee, energy drinks, and some soft drinks), alcohol, medication, and certain foods that can be difficult to digest, such as fatty meats, certain vegetables and fruits, brown rice, red rice, lard, margarine, fatty fish, fatty sashimi, and cheese for 24 h (Yamashita and Yamamoto 2021). On the test day, the children were asked to empty their bladder upon arrival at the laboratory. They then rested for 30 min, after which a fresh spot urine sample was collected between 09:30 and 15:30. Urine samples were diluted with 6.7 mM hydrochloric acid and 2.5% perchloric acid to separate albumin, as previously reported (Yamashita and Yamamoto 2021). Obtained supernatants were stored at -80 °C until high-performance liquid chromatography (HPLC) was performed.

Urinary MHPG, 5-HIAA, and HVA (Sigma-Aldrich Inc., St Louis, MO, USA) content was measured using an HPLC device with an electrochemical detector (Nanospace SI-2 3005, Osaka Soda Corporation, Osaka, Japan) and a chromate recorder (C-R8A; Shimadzu Corporation, Kyoto, Japan). The mobile phase consisted of 15% methanol in a solution (pH 4.13) containing 30 mM citric acid, 10 mM disodium hydrogen phosphate, 0.5 mM sodium octyl sulfate, 50 mM sodium chloride, and 0.05 mM ethylenediaminetetraacetic acid, as previously reported (Yamashita and Yamamoto 2017, 2021). This was pumped through a 5-µM C_18_ column (150 mm × 4.6 mm; TSK gel, ODS-80TM, Tosoh, Tokyo, Japan) at a flow rate of 0.7 mL/min on a single pump (Nanospace SI-2 3101; Osaka Soda Corp., Osaka, Japan) and temperature of 25 °C in the column oven (Nanospace SI-I 2004; Shiseido Corporation, Tokyo, Japan). MHPG, 5-HIAA, and HVA levels were detected electrochemically (voltage: 700 mV) with retention times of approximately 6, 12, and 18 min, respectively. Urine MM levels were calculated from HPLC-derived peak values after accounting for dilution factors and urine collection conditions, including the fixed 30-min collection interval. These values were expressed as normalized urinary MM levels for subsequent analysis.

### Statistical analysis of demographic, psychological, cognitive, and MMs data

Statistical analyses were conducted using R (version 4.3.0; The R Foundation for Statistical Computing, Vienna, Austria), except for voxel-based morphometry (VBM) statistical analysis. All data were initially assessed for normality and homogeneity of variance, and statistical tests were determined accordingly. Differences between groups based on demographic and behavioral symptoms data were analyzed using Student’s *t*-test, Welch’s *t*-test, Chi-square test, Fisher’s exact test, one-way analysis of variance (ANOVA), and Welch’s one-way ANOVA.

MM data were analyzed using unsupervised clustering to identify NDD subtypes, following previous studies that classified patient subtypes based on molecular and brain functions (Williams et al. 2023; Yu et al. 2022). This unsupervised approach was selected to discover latent transdiagnostic subgroups from urinary MM profiles without stratifying the clustering by diagnosis. Moreover, consistent with previously reported approaches (Scheerer et al. 2021; Yu et al. 2022), cluster analysis was restricted to participants with NDDs, because the primary purpose of this study was to identify monoamine-based subtypes within the clinical group. TD participants were not included in the clustering step to avoid deriving clusters driven primarily by broad case-control differences; instead, they were used as an independent control group for downstream comparisons to characterize the identified subtypes. MM levels were winsorized at three standard deviations from the mean to reduce the influence of outliers and were then converted to z-scores to mitigate distance-associated issues in cluster analysis. The NbClust R package (Charrad et al. 2014) was used to determine the optimal number of clusters using the majority rule across multiple cluster validity indices (e.g., the Silhouette, Duda, and Beale indices), whereby the number of clusters supported by the largest number of indices was selected as the best-fitting solution. Accordingly, NbClust was run using the Euclidean distance and K-means method to partition NDD participants into the optimal number of clusters based on their urinary MM profiles. Furthermore, a support vector machine (SVM) with a radial basis function kernel (R packages “e1071” and “caret”) was used to assess the internal reliability and reproducibility of subtyping based on MM patterns. We applied five-fold cross-validation and three repeats to mitigate the potential risks of overfitting, and the procedure was repeated 100 times for a training dataset (70% of the 83 participants with NDD, *n* = 58) and a test dataset (30% of the 83 participants with NDD, *n* = 25). Model performance was assessed using accuracy, precision, recall, and F1-scores.

We assessed MM patterns by comparing each identified NDD subtype with the TD group using a linear mixed-effects model (R package “lmerTest,” “MuMIn,” and “jtools”), with each MM level set as the dependent variable and group (e.g., NDD-A vs. TD) set as the independent variable. To account for non-independence of observations within families (e.g., among siblings), the family ID was modeled as a random intercept. Covariates included demographic variables (e.g., age and sex; Online Resource 2) that showed significant differences between each NDD subtype group and the TD group. Moreover, based on the biochemical and psychological differences between groups, we investigated the associations between MM levels and psychological characteristics using Spearman’s rank-order correlation coefficients (R package “psych”). For linear mixed-effects models, the statistical threshold was set at *p* < 0.05, false discovery rate (FDR)-corrected using the Benjamini-Hochberg method. Thereafter, the statistical threshold for multiple group comparisons was set at *p* < 0.05, family-wise error (FWE)-corrected using the Bonferroni method. For correlation analyses, the statistical threshold was set at *p* < 0.05, FDR-corrected using the Benjamini–Hochberg method.

We evaluated cognitive features by comparing each NDD subtype group with the TD group by using a linear mixed-effects model, with each cognitive function set as the dependent variable and group set as the independent variable. Random intercepts, covariates, and statistical thresholds were the same as those employed to assess MM levels. Moreover, correlation analyses (only for MM levels and cognitive performances where group differences were significant) were conducted by calculating Spearman’s rank-order correlation coefficients. The statistical threshold was the same as that employed to assess the associations between MM levels and psychological characteristics.

### Image acquisition

Scanning was performed using a 3T GE Signa PET/MR scanner (General Electric Healthcare, Chicago, IL, USA). Children’s heads were immobilized using a head-coil scanner (eight channels). High-resolution structural images were acquired using an axial T1-weighted magnetization-prepared rapid gradient-echo pulse sequence (TR = 8.5 ms; TE = 3.2 ms; field of view = 256 × 256; matrix size = 256 × 256; voxel size = 1 × 1 × 1 mm; 176 slices). Prior to processing, an experienced rater carefully checked the structural scans for artifacts.

### Image preprocessing and statistical analysis

The MRI data were analyzed using VBM, employing Statistical Parametric Mapping 12 (SPM12) software (Wellcome Department of Cognitive Neurology, London, UK), and surface-based morphometry, employing FreeSurfer (version 7.3.0) methods, to assess structural brain differences comprehensively. VBM quantifies regional GMVs across the whole brain, whereas surface-based morphometry focuses on the cortical surface area, reflecting cortical expansion and neurodevelopmental variations. Combining these methods enables a more detailed characterization of brain morphology.

VBM (Ashburner and Friston 2000) was performed using SPM12 software running in MATLAB R2021a. This method was used to pre-process T1-weighted images and calculate the GMV across the whole brain. Structural T1-weighted images were first segmented to separate different types of tissues: gray matter, white matter, cerebrospinal fluid, soft tissue, and the skull. The images (gray matter and white matter) were then spatially normalized using diffeomorphic anatomical registration with exponentiated Lie algebra. To preserve the absolute GMV, normalized gray matter images were modulated by multiplying the Jacobian determinants derived from spatial normalization. Finally, the modulated gray matter images were smoothed with an 8-mm full-width at half-maximum Gaussian kernel. For comparison of VBM between each NDD subtype group and the TD group, we used threshold-free cluster enhancement (TFCE), which was introduced to enhance voxel-based analysis sensitivity by applying the Freedman–Lane method based on 5000 permutations (Freedman and Lane 1983; Winkler et al. 2014). The nuisance variable included total intracranial volume. The covariates were the same as employed to assess the MM levels, in addition to handedness and total intracranial volume. Moreover, an explicit mask was used to exclude noisy voxels from statistical analysis. Based on the TFCE analysis, statistical significance was set at an FWE-corrected threshold of *p* < 0.025 for multiple group comparisons (e.g., NDD-A vs. TD and NDD-B vs. TD). Thereafter, we identified the cluster locations obtained from the structural data using the anatomy toolbox in SPM12.

Next, associations between MM levels and GMV in regions of interests (ROIs) were investigated. Based on the difference in GMVs between groups, GMV values were extracted from the local maxima of significant clusters identified in the SPM analysis. To ensure consistency with the group-level model, covariate-adjusted GMV values were calculated in MATLAB by removing the effects of covariates included in the model. Correlation analyses were conducted using the Spearman’s rank-order correlation coefficients. The statistical threshold was the same as that employed to assess the associations between MM levels and psychological characteristics.

FreeSurfer was used to reconstruct the brain cortical surface from T1-weighted images, which automates the quantitative assessment of brain anatomy (Fischl et al. 2002). Details of the segmentation procedures have been described previously (Dale et al. 1999; Fischl et al. 2002). Finally, we used 34 regions labeled with the Desikan atlas-based classification for the cortical surface area (68 regions in total). To compare regional cortical surface areas between each NDD subtype group and the TD group, we adopted a linear mixed-effects model with each regional cortical surface area set as the dependent variable and group set as the independent variable. Random intercepts, covariates (in addition to handedness and total intracranial volume), and statistical thresholds were the same as those employed to assess the MM levels. Moreover, based on the biochemical and surface area differences between groups, we investigated the associations between MM levels and regional cortical surface areas using Spearman’s rank-order correlation coefficients. The statistical threshold was the same as that employed to assess the associations between MM levels and psychological characteristics.

## Results

### Demographics and MM pattern clustering

The demographic data are shown in Table 1. Student’s *t*-tests showed significant differences between the TD and NDD groups in terms of age, years of education, annual household income, and intelligence quotient. Chi-square tests showed significant group differences in sex ratio and assessment season distribution.

**Fig. 1:**
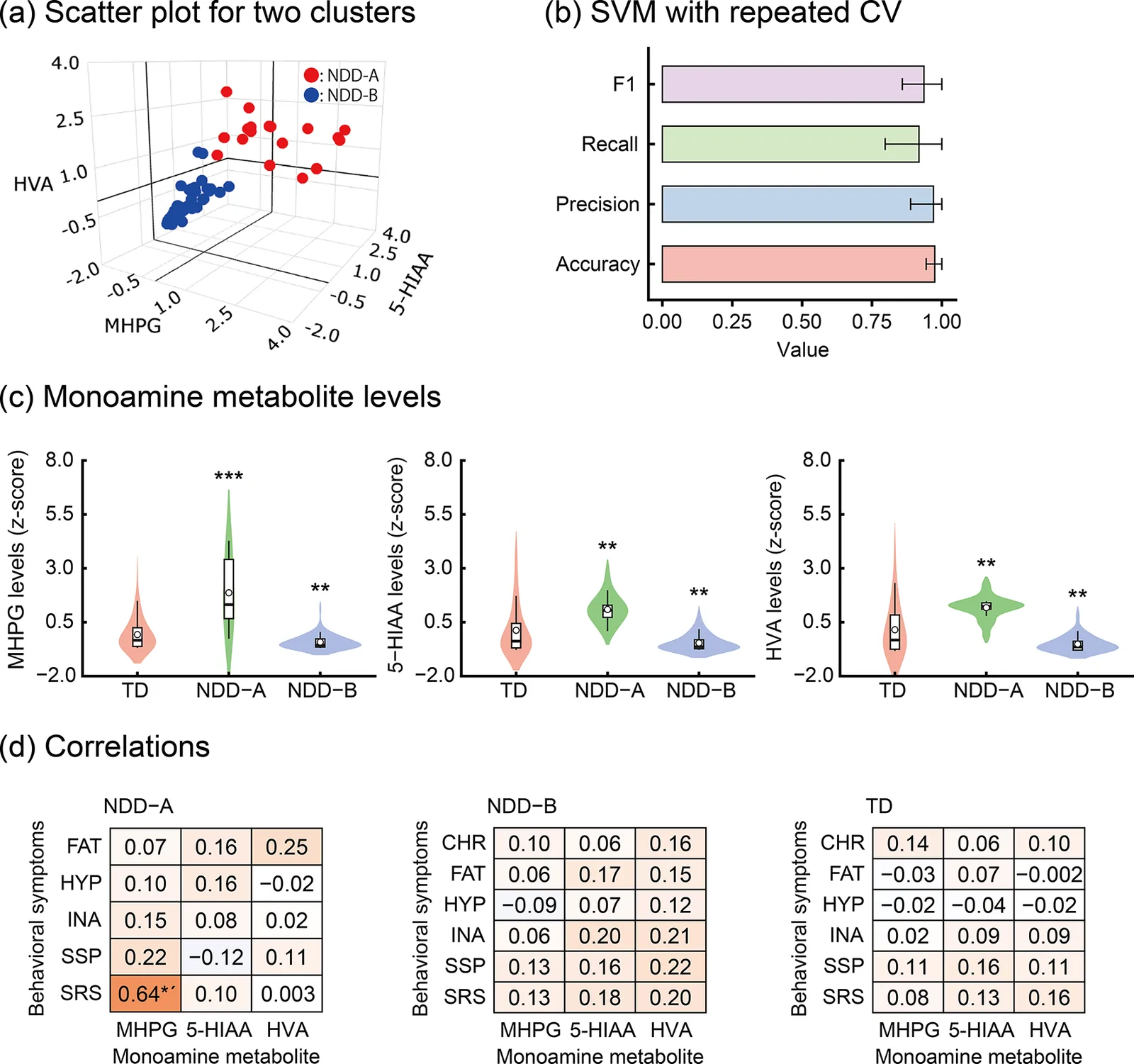
Neurodevelopmental disorder subtypes based on urinary monoamine metabolite patterns. **a** Using NbClust with the Euclidean distance and K-means method, a two-cluster solution was selected by majority rule across cluster validity indices. The scatter plot shows z-scored urinary MHPG, 5-HIAA, and HVA levels, colored by cluster assignment. **b** The SVM algorithm with repeated cross-validation, designed to mitigate the potential risk of overfitting, showed high classification performance. **c** Based on FDR- and FWE-corrected thresholds (*p* < 0.05), the NDD-A subtype showed higher MHPG, 5-HIAA, and HVA levels than those of the TD group. The NDD-B subtype showed lower MHPG, 5-HIAA, and HVA levels than those of the TD group. **d** In the associations between monoamine metabolite levels and behavioral symptoms, the NDD-A subtype displayed a positive correlation between MHPG levels and SRS-2 total score. In the NDD-B subtype and TD group, such correlations were not significant. *** *p*-FWE < 0.001, ** *p*-FWE-*p* < 0.01, *´ *p*-FDR < 0.05. *5-HIAA* 5-hydroxyindoleacetic acid, *CHR* chronotype, *FAT* fatigue, *FDR* false discovery rate, *FWE* family-wise error, *HVA* homovanillic acid, *MHPG* 4-hydroxy-3-methoxyphenylglycol, *NDD* neurodevelopmental disorder, *SSP* short sensory profile, *SRS* social responsiveness scale, *SVM* support vector machine, *TD* typical development

Using NbClust with the Euclidean distance and K-means method in the NDD sample (*n* = 83), 11 of the 26 validity indices supported a two-cluster solution. Based on the majority rule, k = 2 was selected as the optimal clustering solution. Figure 1a presents a scatter plot of z-scored urinary MHPG, 5-HIAA, and HVA levels for NDD participants, illustrating a two-cluster solution. These NDD subtypes were named NDD-A (*n* = 18) and NDD-B (*n* = 65). To assess the internal reliability and reproducibility of these subtypes, the SVM algorithm with repeated cross-validation showed high accuracy (0.97 ± 0.03), precision (0.97 ± 0.08), recall (0.92 ± 0.12), and F1 scores (0.94 ± 0.08) in the test dataset (Fig. 1b), suggesting that the two subtypes were distinguishable based on urinary MM profiles. The demographics and behavioral symptoms of each NDD subtype are shown in Online Resource 2.

MM data are shown in Fig. 1c. The main effect of group in the linear mixed-effects model showed that the NDD-A subtype had significantly higher levels of MHPG (*β* = 1.84, 95% CI [1.39, 2.29], *R*^2^ = 0.43, *t* = 7.99, *p*-FDR < 0.001, *p*-FWE < 0.001), 5-HIAA (*β* = 0.91, 95% CI [0.36, 1.45], *R*^2^ = 0.11, *t* = 3.27, *p*-FDR = 0.001, *p*-FWE = 0.002), and HVA (*β* = 1.00, 95% CI [0.46, 1.54], *R*^2^ = 0.13, *t* = 3.63, *p*-FDR < 0.001, *p*-FWE = 0.001) than the TD group. Moreover, the main effect of group indicated that the NDD-B subtype had significantly lower levels of MHPG (*β* = -0.62, 95% CI [-0.99, -0.24], *R*^2^ = 0.09, *t* = 3.27, *p*-FDR = 0.001, *p*-FWE = 0.003), 5-HIAA (*β* = -0.58, 95% CI [-0.95, -0.22], *R*^2^ = 0.11, *t* = 3.14, *p*-FDR = 0.002, *p*-FWE = 0.004), and HVA (*β* = -0.65, 95% CI [-1.01, -0.29], *R*^2^ = 0.14, *t* = 3.55, *p*-FDR = 0.001, *p*-FWE = 0.003) than the TD group. These results revealed distinct monoaminergic activity in the NDD subtypes, with hyperactivity in NDD-A and hypoactivity in NDD-B.

Subsequently, we investigated associations between MM levels and psychological measures (Fig. 1d). In the NDD-A subtype, MHPG levels were positively correlated with the SRS-2 total score (*p*-FDR = 0.013). By contrast, the NDD-B subtype and TD group did not show correlations between MM levels and psychological measures. These results indicate that in the NDD-A subtype, elevated noradrenergic activity is linked to difficulties in social communication.

### Cognitive characteristics

**Fig. 2:**
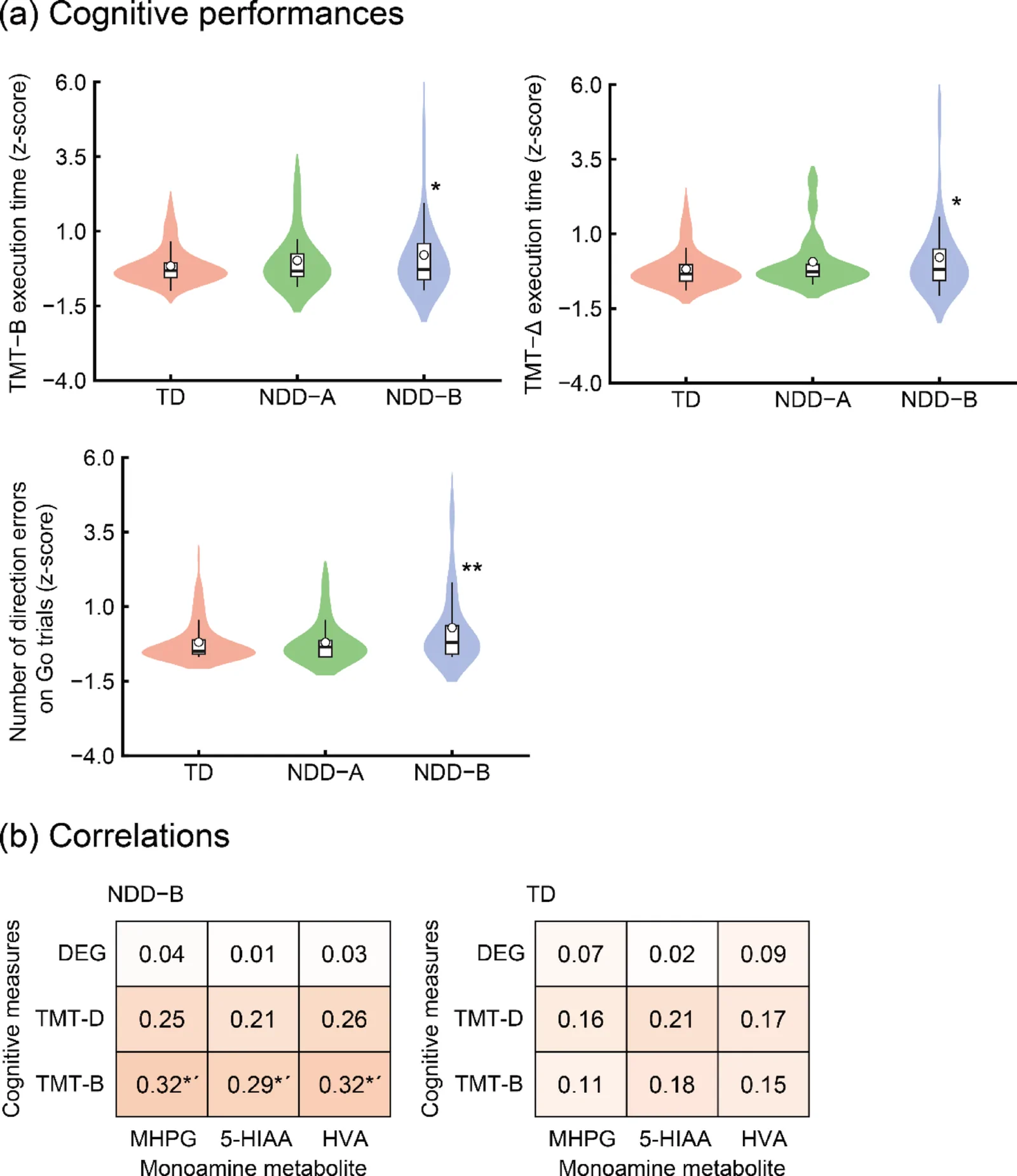
Cognitive differences between the groups. **a** Based on FDR- and FWE-corrected thresholds (*p* < 0.05), the NDD-B subtype showed worse performance in the TMT-B, TMT-Δ, and direction errors on the go trial of the stop signal task as compared with the TD group. **b** In the NDD-B subtype, levels of MHPG, 5-HIAA, and HVA were positively correlated with the time taken to complete the TMT-B. In contrast, these correlations with monoamine metabolites were not significant in the TD group. ** *p*-FWE < 0.01, *´ *p*-FDR < 0.05. *5-HIAA* 5-hydroxyindoleacetic acid, *FDR* false discovery rate, *FWE* family-wise error, *HVA* homovanillic acid, *MHPG* 4-hydroxy-3-methoxyphenylglycol, *NDD* neurodevelopmental disorder, *TD* typical development, *TMT* trail-making test

Cognitive data are shown in Fig. 2. The main effect of group in the linear mixed-effect model showed that the NDD-B subtype had significantly greater values for execution time on the TMT-B (*β* = 0.45, 95% CI [0.13, 0.78], *R*^2^ = 0.36, *t* = 2.74, *p*-FDR = 0.015, *p*-FWE = 0.031) and TMT-Δ (*β* = 0.46, 95% CI [0.11, 0.81], *R*^2^ = 0.28, *t* = 2.60, *p*-FDR = 0.015, *p*-FWE = 0.031) as well as the frequency of direction errors on go trials in the stop signal task (*β* = 0.58, 95% CI [0.23, 0.93], *R*^2^ = 0.27, *t* = 3.25, *p*-FDR = 0.004, *p*-FWE = 0.008) than those of the TD group. These results indicates that the NDD-B subtype had lower levels of cognitive control, cognitive flexibility, and inhibitory control. For comparisons of each NDD subtype with the TD group, excluding the abovementioned cognitive performance aspect, see Online Resource 3.

We also investigated the correlation between MM levels and cognitive performance (Fig. 2b). MM levels and execution time on the TMT-B were positively correlated in the NDD-B subtype (MHPG: *p*-FDR = 0.016; 5-HIAA: *p*-FDR = 0.019; HVA: *p*-FDR = 0.016). However, the TD group did not show this correlation. These results indicate that in the NDD-B subtype, enhancement of monoaminergic activity is linked to slower cognitive control.

### Brain structural characteristics

**Fig 3.**
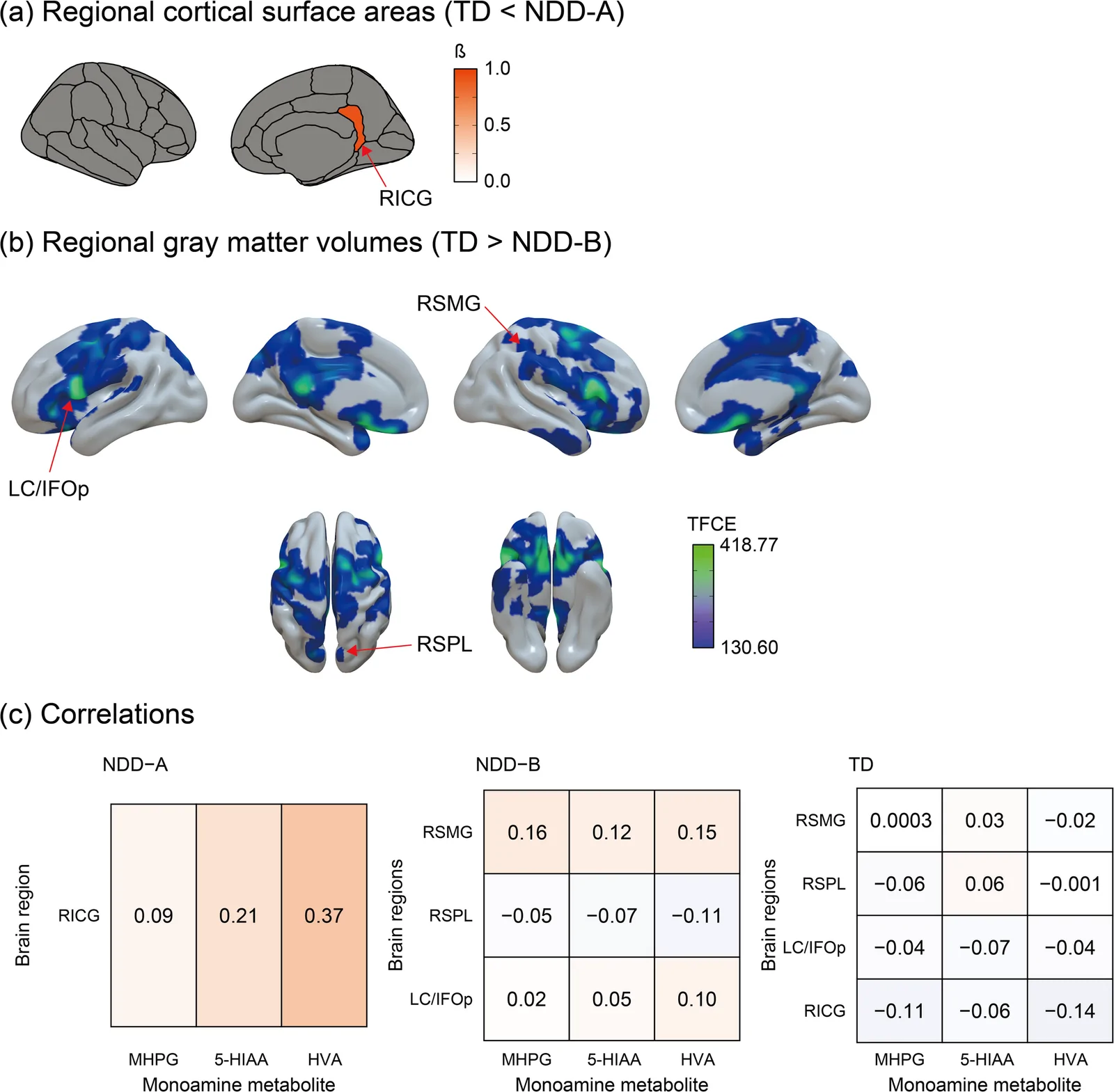
Structural brain differences among the groups. **a** The NDD-A subtype showed a larger surface area in the right isthmus cingulate gyrus than that in the TD group. **b** The NDD-B subtype showed smaller volumes in the left central and inferior frontal opercular regions, right superior parietal lobule, and right supramarginal gyrus than those in the TD group. **c** Using ROIs (where each subtype showed a greater surface area or lesser GMV than that in the TD group), correlations between monoamine metabolites and brain structure were not significant. *5-HIAA* 5-hydroxyindoleacetic acid, *Cu* cuneus, *GMV* gray matter volume, *HVA* homovanillic acid, *RICG* right isthmus cingulate gyrus, *LC/IFOp* left central/inferior frontal opercular regions, *MHPG* 4-hydroxy-3-methoxyphenylglycol, *NDD* neurodevelopmental disorder, *RSMG* right supramarginal gyrus, *RSPL* right superior parietal lobule, *TD* typical development, TFCE threshold-free cluster enhancement

Brain structural data are shown in Fig. 3. The main effect of group in the linear mixed-effects model showed that the NDD-A subtype exhibited a larger surface area in the right isthmus cingulate gyrus (*β* = 1.02, 95% CI [0.49, 1.56], *R*^2^ = 0.29, *t* = 3.74, *p*-FDR = 0.022, *p*-FWE = 0.045) than the TD group. However, the NDD-B subtype did not show significant differences in regional cortical surface areas. Additionally, whole-brain voxel-based GMV analysis using TFCE revealed a large significant cluster extending across fronto–opercular and orbitofrontal regions when comparing the NDD-B subtype with the TD group (Online Resource 4). The primary peak within this cluster indicated smaller volumes in the left central and inferior frontal opercular regions in the NDD-B subtype than in the TD group. Additional significant peaks revealed reduced volumes in the right superior parietal lobule and right supramarginal gyrus in the NDD-B subtype than in the TD group. In contrast, the NDD-A subtype did not exhibit significant differences in regional brain volumes. These results highlight the distinct patterns of structural brain differences between the NDD subtypes. Subsequently, we investigated the associations between MM levels and brain structural characteristics (where each NDD subtype showed greater surface or lesser GMV than did the TD group) (Fig. 3c); no correlations were found between MM levels and brain structural characteristics.

## Discussion

This study aimed to identify urinary MM-based NDD subtypes across ADHD and ASD using an unsupervised clustering approach and to examine structural brain characteristics associated with these subtypes. Cognitive performance was also examined to inform the interpretation of the identified subtypes. Clustering revealed two distinct NDD subtypes: NDD-A, characterized by high MHPG, 5-HIAA, and HVA levels in urine; and NDD-B, characterized by low levels of these three MMs. Although no significant differences in behavioral symptoms were observed between the two NDD subtypes, both subtypes showed greater difficulties in social communication and sensory processing, greater severity of inattention and hyperactivity/impulsivity, and greater fatigue than the TD group (Online Resource 2). Greater difficulty in social communication was associated with higher levels of MHPG in the NDD-A subtype. Moreover, compared with the TD group, only the NDD-B subtype showed reduced performance in cognitive control, cognitive flexibility, and inhibitory control. Notably, compared with the TD group, the cortical surface areas were larger in the isthmus cingulate gyrus in the NDD-A subtype, whereas the NDD-B subtype exhibited smaller GMV primarily in fronto–opercular/orbitofrontal regions, with additional reductions in the right superior parietal lobule and right supramarginal gyrus. These findings suggest that the NDD-A and NDD-B subtypes are characterized by distinct monoaminergic profiles and structural brain differences. However, because urinary MMs may reflect both central and peripheral monoaminergic metabolism, these subtypes should be interpreted as urinary MM-defined monoaminergic profiles, rather than as direct indicators of brain monoamine activity.

### Specificity of monoamine hyperfunction and hypofunction subtypes

We found that the urinary levels of MHPG, 5-HIAA, and HVA were higher in the NDD-A than in the TD group. This urinary MM profile provides indirect support for the hypothesis of monoaminergic hyperactivity in NDDs (Chugani et al. 1999; Dvoráková et al. 2007; Goldberg et al. 2009; Ulke et al. 2019). Notably, the NDD-A subtype displayed a positive correlation between urinary MHPG levels and social communication difficulties, whereas no significant differences in cognitive function were found between the NDD-A and TD groups. A relevant concept for interpreting this finding is the inverted U-shaped relationship between monoamine levels and cognitive function (Arnsten 2009; Baldi and Bucherelli 2005). This framework may help explain why NDD-A showed an association between higher urinary MHPG levels and social communication difficulties, despite showing no significant cognitive impairment relative to the TD group.

The noradrenergic pathway projects from the locus coeruleus to the amygdala and cingulate cortex (Heilbronner and Haber 2014; Joshi et al. 2016; Stahl 2021). A pharmacological study in mice reported that administration of a selective noradrenergic neurotoxin reduced noradrenergic activity in the locus coeruleus, increased anxiety-related social behavior, and decreased noradrenaline content in the amygdala (Itoi et al. 2011), suggesting a role for the locus coeruleus–amygdala noradrenergic circuit in social behavioral regulation. Moreover, Monk et al. (2010) revealed stronger connectivity between the amygdala and ventral anterior cingulate cortex in individuals with ASD than in TD controls during the viewing of faces depicting socially relevant emotions, suggesting atypical processing of social-emotional information. Accordingly, relatively increased noradrenergic metabolism, potentially involving the locus coeruleus–cingulate cortex–amygdala pathway, may contribute to social communication difficulties in NDD-A. Serotonergic mechanisms may also be involved in social communication difficulties (Kheirouri et al. 2016; Yang et al. 2015). Considering that noradrenaline facilitates serotonin efflux by binding to the α1 receptor on serotonergic neurons (Stahl 2021), interactions between the noradrenergic and serotonergic systems may contribute to social communication impairment in NDD-A. Nevertheless, the observed association between urinary MHPG and social communication difficulties suggests that the noradrenergic component may be particularly relevant.

The lower urinary MM levels observed in NDD-B may be consistent with previous studies suggesting monoaminergic hypoactivity in NDDs (Alabdali et al. 2014; Gevi et al. 2016, 2020; Hannestad et al. 2010; Hellings 2023; Mechler et al. 2022; Ota et al. 2021). The NDD-B subtype also showed poorer cognitive control, cognitive flexibility, and inhibitory control than the TD group, suggesting that executive function difficulties in this subtype may be interpreted within the context of a urinary MM-defined profile of relatively reduced monoaminergic metabolism. However, higher urinary MM levels were associated with reduced cognitive control in the NDD-B subtype. Because the TMT-B performance is also associated with frontal executive function (Chan et al. 2015), this finding should be interpreted in conjunction with other measures of executive function when assessing cognitive control.

The serotonergic system projects from the raphe nuclei to the prefrontal cortex via the ventral tegmental area and locus coeruleus (Hensler 2006; Stahl 2021). In addition, the prefrontal cortex receives direct projections from dopaminergic neurons in the ventral tegmental area and noradrenergic neurons in the locus coeruleus (Arnsten et al. 2015; Aston-Jones and Cohen 2005; Stahl 2021). Moreover, serotonin binding to 5-HT2A receptors on noradrenergic neurons in the locus coeruleus and dopaminergic neurons in the ventral tegmental area can regulate the release of noradrenaline and dopamine into the prefrontal cortex (Stahl 2021), highlighting interactions among serotonergic, noradrenergic, and dopaminergic systems in prefrontal cortical regulation. Through this modulation, monoaminergic activity may be related to executive function. Neuroimaging and pharmacological studies have reported that serotonergic, noradrenergic, and dopaminergic activity modulates executive function, such as cognitive control and set shifting (Arnsten and Li 2005; Arnsten and Pliszka 2011; Kuwamizu et al. 2023; Yamashita and Yamamoto 2021). These findings suggest that NDD-B may involve relatively reduced monoaminergic regulation associated with executive function impairments, particularly those related to prefrontal cortical function.

Taken together, the contrasting urinary MM-defined profiles of NDD-A and NDD-B suggest that monoaminergic heterogeneity may be differentially associated with social communication difficulties in NDD-A and executive function impairments in NDD-B. These findings highlight the potential value of subtype-specific approaches to assessment and treatment in NDDs.

### Distinct brain structural anomalies in monoamine hyperfunction and hypofunction subtypes

Surface area enlargement in the NDD-A subtype was observed in the right isthmus cingulate gyrus. Anatomically, the isthmus cingulate is a key structure that connects the posterior cingulate cortex to the parahippocampal gyrus (Caeyenberghs et al. 2016; Chen et al. 2021). Although not well understood, the isthmus cingulate gyrus may be involved in emotional regulation and episodic memory processing, based on its association with anatomical connections (Weerasekera et al. 2024). Interestingly, a greater surface area in the isthmus cingulate gyrus has been associated with worse social ability scores in individuals with ASD (Doyle-Thomas et al. 2013), suggesting the involvement of atypical isthmus cingulate gyrus morphometry in social behavioral impairments in NDDs. Studies on ASD have shown associations between an increase in cortical surface area and cerebral cortex overgrowth (Donovan and Basson 2017; Ohta et al. 2016), suggesting that an increased cortical surface area, possibly linked to abnormal gyrification (Kitajima et al. 2023), may lead to neuronal overgeneration and abnormal neural connections (Hogstrom et al. 2013; Meyer et al. 2014). Furthermore, the cingulate cortex, including the isthmus cingulate gyrus, is a key target of the ascending noradrenergic pathway originating from the locus coeruleus (Heilbronner and Haber 2014; Joshi et al. 2016; Stahl 2021), suggesting that the noradrenergic activity in the locus coeruleus–cingulate cortex circuit, together with the amygdala, may affect social functional impairments through its modulation of emotional regulation (Itoi et al. 2011; Monk et al. 2010; Terbeck et al. 2016). Our findings suggest that surface area enlargement in the isthmus cingulate gyrus may be a key factor in the social communication difficulties observed in individuals with the NDD-A subtype, potentially due to alterations in noradrenergic neural connections.

In contrast to the NDD-A subtype, the NDD-B subtype showed smaller GMV primarily in a large fronto–opercular cluster with orbitofrontal involvement, and the largest peak was located in the left central and inferior frontal opercular regions. This structural finding is consistent with a recent meta-analysis showing reduced GMV in the left inferior frontal gyrus, including its opercular and orbital parts, in ADHD (Yu et al. 2023), suggesting that NDD-B may involve ADHD-related fronto–opercular structural characteristics. Moreover, Depue et al. (2010) reported that a reduction in GMV of the inferior frontal gyrus, particularly in the pars opercularis, was associated with poorer response inhibition in individuals with combined-type ADHD. In addition, the orbitofrontal involvement observed in the large cluster is notable because the orbitofrontal cortex contributes to executive functioning processes together with other frontal areas (Deng et al. 2017; Rolls 2019). Thus, structural diminishment within fronto–opercular/orbitofrontal regions may help explain the executive control difficulties observed in NDD-B. Furthermore, serotonergic regulation of frontal cortical systems may provide a molecular context for this interpretation, given evidence linking serotonin transporter-related vulnerability under psychosocial stress to frontal GMV reductions and ADHD symptom severity (van der Meer et al. 2015). Serotonin can also modulate noradrenaline and dopamine efflux via 5-HT2A receptor activity in the frontal cortex (Bortolozzi et al. 2005; Stahl 2021). These findings suggest that the fronto–opercular/orbitofrontal structural features and executive control difficulties observed in NDD-B may be interpreted within the context of relatively reduced monoaminergic regulation.

In addition to the fronto–opercular/orbitofrontal regions, the NDD-B subtype showed smaller GMV in the right superior parietal lobule and right supramarginal gyrus than the TD group. The right superior parietal lobule is important for visuospatial attention and top-down attentional processes (Banaszkiewicz et al. 2021; Zhang et al. 2025). The right supramarginal gyrus has also been implicated in speech production (Peng et al. 2025). These additional parietal findings may suggest localized vulnerability in attention and language-related processes in NDD-B, beyond executive control processes.

Taken together, the structural findings indicate subtype-specific neuroanatomical profiles in urinary MM-defined NDD subtypes: NDD-A showed surface area enlargement in the isthmus cingulate gyrus, whereas NDD-B exhibited GMV reductions primarily in fronto–opercular/orbitofrontal regions, with additional reductions in the superior parietal lobule and supramarginal gyrus.

### Clinical implications and limitations

The National Institute of Mental Health in the United States launched the Research Domain Criteria projects to establish a novel framework for pathophysiological research (Insel and Cuthbert 2015). In line with this framework, the present study examined urinary MM patterns categorized using unsupervised machine learning and the associated brain structural characteristics in individuals with ADHD and/or ASD. Despite clear differences in urinary MM profiles, behavioral symptoms did not differ between NDD subtype groups; these could possibly be caused by other molecular and neural mechanisms. Additionally, our findings highlight the potential importance of personalized therapeutic strategies and careful reconsideration of pharmacological interventions. Because NDD-A showed a urinary MM-defined profile of relatively increased monoaminergic metabolism, treatments that further enhance monoaminergic signaling, such as methylphenidate, atomoxetine, or serotonin reuptake inhibitors, may require careful consideration rather than uniform application. Another potential approach may involve branched-chain amino acids (BCAAs), given their role in regulating serotonin and its precursor tryptophan (Yamashita 2020). Because BCAAs share the system L transporter at the blood–brain barrier with tryptophan, they may influence serotonin synthesis by regulating tryptophan availability in the brain (Yamashita 2020). As naturally occurring amino acids found in food and supplements, BCAAs may represent a potential non-pharmacological approach that warrants further investigation.

Our study had some limitations. First, our clustering results might be considered preliminary due to the small sample size for NDD-A, which limited the statistical power and generalizability of the results of the study. Second, the cross-sectional design precluded conclusions about causal relationships. Third, urinary MM levels were calculated as normalized urinary MM levels based on dilution factors and urine collection conditions, including the fixed 30-min collection interval, but they were not corrected using creatinine or specific gravity. Therefore, residual effects of hydration-related variability cannot be completely excluded. Forth, the present study could not determine the relative contributions of central and peripheral sources to urinary MM levels. Although urinary MM may partly reflect central monoaminergic metabolism (Edwards et al. 1985; Morinaga et al. 2017), these metabolites may also be influenced by peripheral monoaminergic activity.

## Conclusions

We identified NDD subtypes among children with ADHD and/or ASD using noninvasive urinary MM profiles and examined their neurobiological characteristics while accounting for various confounding factors. To our knowledge, this approach has not previously been described in this field. The urinary MM-defined NDD-A and NDD-B subtypes showed distinct monoaminergic profiles and subtype-specific neuroanatomical profiles. This study presents a novel framework for understanding within-disorder heterogeneity and cross-disorder phenotypic overlaps in ADHD and ASD.

## Supplementary Information

Below is the link to the electronic supplementary material.

Supplementary Material 1

Supplementary Material 2

Supplementary Material 3

Supplementary Material 4

## Data Availability

The data will be made available via the Child Developmental MRI (CDM) Project database, which is currently under construction. Additionally, data will be provided upon signing a data sharing agreement and after receiving a research proposal along with evidence of approval from the requester’s institutional review board.
