# Supplementary material for: Transdiagnostic monoamine-based subtyping for attention-deficit/hyperactivity disorder and autism spectrum disorder via unsupervised machine learning
Source: J Neural Transm (Vienna). 2026 Jul 27;133(8):1937–52. doi: 10.1007/s00702-026-03206-z (PMC13545128; doi:10.1007/s00702-026-03206-z)
Supplement: Supplementary file 1 [file 702_2026_3206_MOESM1_ESM.docx]

**Journal of Neural Transmission**

**Transdiagnostic monoamine-based subtyping for attention-deficit/hyperactivity disorder and autism spectrum disorder via unsupervised machine learning**

Masatoshi Yamashita^*^, Qiulu Shou, Sayo Hamatani, Hideo Matsuzaki, Masanori Fujieda, Yoshiyuki Hirano, Kuriko Kagitani-Shimono, Hidehiko Okazawa, and Yoshifumi Mizuno^#^

***Corresponding author**

Masatoshi Yamashita, Research Center for Child Mental Development, Hamamatsu University School of Medicine, Shizuoka, Japan, email: ymasa09@hama-med.ac.jp

**^#^Co-corresponding author**

Yoshifumi Mizuno, Research Center for Child Mental Development, University of Fukui, Fukui, Japan, email: mizunoy@u-fukui.ac.jp

**Online Resource 1: The details of psychological questionnaire**

The Social Responsiveness Scale (SRS-2) (Constantino 2021) is a 65-item questionnaire used to measure the severity of social difficulties associated with autism on a 4-point Likert scale (1 = not at all to 4 = very). T-total scores were assessed, with higher scores representing greater severity. The Short Sensory Profile (SSP) (McIntosh et al. 1999) is a 38-item questionnaire used to measure sensory atypicality associated with autism on a 5-point Likert scale (1 = not at all to 5 = very). The total T-scores were assessed, with higher scores representing sensory abnormalities. Conners 3 is a 108-item questionnaire for parents and a 99-item questionnaire for children that measures the presence of the most prominent symptoms of attention-deficit/hyperactivity disorder (ADHD) on a 4-point Likert scale (0 = not at all to 3 = very). The T-scores were used to assess inattention and hyperactivity/impulsivity. The Chalder fatigue scale (Chalder et al. 1993) is a 14-item questionnaire used to measure physical and mental fatigue on a 4-point Likert scale (0 = less than usual to 3 = much more than usual); total scores were evaluated. The Munich Chronotype Questionnaire (Roenneberg et al. 2003) is a standardized self-rating scale used to assess an individual’s habitual sleep and wake times on school and free days. The midpoint of sleep on free days corrected for sleep debt (MSFsc) was used as a chronotype index. MSFsc is calculated as the midpoint of sleep on free days minus a correction for sleep debt equal to half the difference between sleep duration on free days and the average sleep duration over the week.

**Online Resource 1: The details of cognitive function task**

The stop signal task (Chen et al. 2016; Rhodes et al. 2012) was conducted to assess inhibition response. Children were asked to quickly respond to an arrow stimulus by selecting one of two options depending on the direction in which the arrow was pointing. Thereafter, children were instructed to withhold their behavioral responses when an auditory signal was present. We used direction errors on stop and go trials, recorded when the children pressed the wrong button. Further, we used the stop signal reaction time, calculated as the mean reaction time minus the stop signal delay. The spatial working memory task (Chen et al. 2016; Rhodes et al. 2012) was conducted to assess the retention of spatial information and the retrieval of retained items from working memory. Children were asked to find blue tokens hidden inside a number of colored boxes on the screen and place them in an empty column on the side of the screen. As the color and position of the boxes were altered to avoid a stereotyped search strategy in each trial, children were instructed not to return to a box where a token was previously found. We used “within errors,” recorded when the children opened an empty box more than twice within a single search sequence. Additionally, we used “between errors,” recorded when the children opened a box where a token was already found in an n-box trial. Parts A and B of the TMT (Reitan and Wolfson 1985) were conducted to evaluate executive control. For TMT-A, children were asked to draw lines, sequentially connecting 25 numbers in ascending order. For TMT-B, children were asked to draw lines alternately between numbers and letters (“1” → “あ” → “2” → “い”, and so on). The time to complete each task was measured. Further, we assessed TMT-Δ, calculated as the time to complete part B minus part A.

References

Chalder T, Berelowitz G, Pawlikowska T, Watts L, Wessely S, Wright D, Wallace EP (1993) Development of a fatigue scale. J Psychosom Res 37: 147-53.

Chen SF, Chien YL, Wu CT, Shang CY, Wu YY, Gau SS (2016) Deficits in executive functions among youths with autism spectrum disorders: an age-stratified analysis. Psychol Med 46: 1625-38.

Constantino JN (2021) Social responsiveness scale Encyclopedia of autism spectrum disorders. Springer, pp 4457-4467

McIntosh D, Miller L, Shyu V, Dunn W (1999) Sensory profile manual. San Antonio, Texas: Psychological Corporation.

Reitan RM, Wolfson D (1985) The Halstead-Reitan neuropsychological test battery: Theory and clinical interpretation. Neuropsychology press

Rhodes SM, Park J, Seth S, Coghill DR (2012) A comprehensive investigation of memory impairment in attention deficit hyperactivity disorder and oppositional defiant disorder. J Child Psychol Psychiatry 53: 128-37.

Roenneberg T, Wirz-Justice A, Merrow M (2003) Life between clocks: daily temporal patterns of human chronotypes. J Biol Rhythms 18: 80-90.
