# Supplementary material for: Transdiagnostic monoamine-based subtyping for attention-deficit/hyperactivity disorder and autism spectrum disorder via unsupervised machine learning
Source: J Neural Transm (Vienna). 2026 Jul 27;133(8):1937–52. doi: 10.1007/s00702-026-03206-z (PMC13545128; doi:10.1007/s00702-026-03206-z)
Supplement: Supplementary file 2 [file 702_2026_3206_MOESM2_ESM.docx]

**Journal of Neural Transmission**

**Transdiagnostic monoamine-based subtyping for attention-deficit/hyperactivity disorder and autism spectrum disorder via unsupervised machine learning**

Masatoshi Yamashita^*^, Qiulu Shou, Sayo Hamatani, Hideo Matsuzaki, Masanori Fujieda, Yoshiyuki Hirano, Kuriko Kagitani-Shimono, Hidehiko Okazawa, and Yoshifumi Mizuno^#^

***Corresponding author**

Masatoshi Yamashita, Research Center for Child Mental Development, Hamamatsu University School of Medicine, Shizuoka, Japan, email: ymasa09@hama-med.ac.jp

**^#^Co-corresponding author**

Yoshifumi Mizuno, Research Center for Child Mental Development, University of Fukui, Fukui, Japan, email: mizunoy@u-fukui.ac.jp

**Online Resource 2: Additional demographics and behavioral symptoms**

The demographic data for each subtype are presented in **Online Resource 2 Table**. Compared to the TD group, the NDD-A subtype showed significant differences in sex ratio, income, and intelligence quotient. Similarly, the NDD-B subtype showed significant differences in age, education, sex ratio, income, intelligence quotient, and the distribution of assessment season.

The behavioral symptoms data are shown in **Online Resource 2 Table**. Welch’s one-way ANOVA showed significant main effects of group on the SRS-2 total score (*F* _[2.00, 42.69]_ = 62.02, *p* < 0.001, generalized η2 = 0.43), SSP total score (*F* _[2.00, 40.51]_ = 38.40, *p* < 0.001, generalized η2 = 0.32), inattention score (*F* _[2.00, 43.14]_ = 40.16, *p* < 0.001, generalized η2 = 0.33), hyperactivity/impulsivity score (*F* _[2.00, 39.81]_ = 49.04, *p* < 0.001, generalized η2 = 0.38), and fatigue score (*F* _[2.00, 43.27]_ = 21.72, *p* < 0.001, generalized η2 = 0.21). The simple main effects analyses revealed that the NDD-A and NDD-B types had higher SRS-2 total, SSP total, inattention, hyperactivity/impulsivity, and fatigue scores than the TD group. However, there were no significant differences between NDD subtypes in these measures. For chronotype, one-way ANOVA showed a significant main effect of group (*F* _[2, 150]_ = 4.61, *p* = 0.011, partial η2 = 0.06). The simple main effect analyses revealed that the NDD-B type had a greater chronotype than the TD group. However, the NDD-A type did not show such significant differences compared to either the TD group or the NDD-B type. These results indicate that although both NDD subtypes had higher levels of difficulties in social communication and sensory processing, inattention, hyperactivity/impulsivity, and fatigue compared to the TD group, their severity did not differ between the NDD subtypes. Moreover, only the NDD-B type showed a delayed chronotype compared to the TD group.

**Online Resource 2 Table.** Demographics and behavioral symptoms of the typical development group and each neurodevelopmental disorder subtype

| Characteristic | TD  (n = 83) | NDD-A  (n = 18) | NDD-B  (n = 65) | *P*-value |
| --- | --- | --- | --- | --- |
| Age (years) | 10.76 (2.97) | 11.39 (2.33) | 11.85 (3.33) | a: 0.400  b: 0.037 |
| Education (years) | 6.34 (2.92) | 7.00 (2.17) | 7.35 (3.27) | a: 0.280  b: 0.048 |
| Sex (n)  Male  Female | 39  44 | 16  2 | 58  7 | a: 0.002  b: < 0.001 |
| Annual household income (JPY) (n) | | | | |
| < 3 000 000  3 000 000-5 000 000  5 000 000-7 000 000  ≥7 000 000 | 6  9  20  48 | 4  5  6  3 | 6  19  11  29 | a: < 0.001  b: 0.032 |
| Diagnosis (n)  ADHD  ASD  ADHD+ASD | NA | 5  2  11 | 16  19  30 | NA |
| Handedness (n)  Right  Left  Ambidextrous | 78  3  2 | 16  2  0 | 59  6  0 | a: 0.476  b: 0.154 |
| Intelligence quotient (score) | 106.54 (12.74) | 92.17 (11.85) | 97.94 (12.79) | a: < 0.001  b: < 0.001 |
| Urine collection time period (n) | | | | |
| Morning  Midday  Afternoon | 31  30  22 | 9  4  5 | 20  27  18 | a: 0.490  b: 0.686 |
| Assessment season (n) | | | | |
| Spring  Summer  Autumn  Winter | 24  28  11  20 | 4  6  5  3 | 13  16  23  13 | a: 0.491  b: 0.016 |
| SRS-2 (score) | 45.90 (7.60) | 61.11 (12.19) | 64.49 (12.45) | a: < 0.001  b: < 0.001  c: 0.650 |
| SSP (score) | 45.98 (8.60) | 64.61 (18.23) | 64.57 (16.87) | a: < 0.001  b: < 0.001  c: > 0.999 |
| Inattention (score) | 47.99 (8.24) | 65.50 (12.45) | 63.02 (13.90) | a: < 0.001  b: < 0.001  c: > 0.999 |
| Hyperactivity/Impulsivity (score) | 44.89 (6.00) | 62.22 (14.04) | 60.40 (12.97) | a: < 0.001  b: < 0.001  c: > 0.999 |
| Fatigue (score) | 4.72 (4.53) | 11.83 (7.03) | 10.12 (6.37) | a: < 0.001  b: < 0.001  c: 0.760 |
| Chronotype (MSFsc, h) | 2.81 (0.92) | 3.21 (1.12) | 3.35 (1.16) | a: 0.460  b: 0.010  c: > 0.999 |

Parameters are indicated as the mean (*SD*) or n. *P*-values for age, education, income, and intelligence quotient are from *t*-tests for the comparison of TD with each NDD subtype. *P*-values for sex ratio is from chi-square tests for the comparison of TD with each NDD subtype. *P*-values for handedness is from Fisher’s exact tests for the comparison of TD with each NDD subtype. *P*-values for assessment season distribution and urine collection time period distribution are from Fisher’s exact tests for the comparison of the TD group with the NDD-A group, and from chi-square tests for the comparison of the TD group with the NDD-B group. *P*-values are from Welch’s one-way analysis of variance with Bonferroni correction for multiple comparisons. a, TD vs. NDD-A; ADHD, attention deficit hyperactivity disorder; ASD, autism spectrum disorder; b, TD vs. NDD-B; c, NDD-A vs. NDD-B; MSFsc, midpoint of sleep on free days, corrected for sleep debt; NA, not applicable; NDD, neurodevelopmental disorder; SD, standard deviation; SSP, Short Sensory Profile; SRS, Social Responsiveness Scale; TD, typical development.
