# Supplementary material for: Transdiagnostic monoamine-based subtyping for attention-deficit/hyperactivity disorder and autism spectrum disorder via unsupervised machine learning
Source: J Neural Transm (Vienna). 2026 Jul 27;133(8):1937–52. doi: 10.1007/s00702-026-03206-z (PMC13545128; doi:10.1007/s00702-026-03206-z)
Supplement: Supplementary file 3 [file 702_2026_3206_MOESM3_ESM.docx]

**Journal of Neural Transmission**

**Transdiagnostic monoamine-based subtyping for attention-deficit/hyperactivity disorder and autism spectrum disorder via unsupervised machine learning**

Masatoshi Yamashita^*^, Qiulu Shou, Sayo Hamatani, Hideo Matsuzaki, Masanori Fujieda, Yoshiyuki Hirano, Kuriko Kagitani-Shimono, Hidehiko Okazawa, and Yoshifumi Mizuno^#^

***Corresponding author**

Masatoshi Yamashita, Research Center for Child Mental Development, Hamamatsu University School of Medicine, Shizuoka, Japan, email: ymasa09@hama-med.ac.jp

**^#^Co-corresponding author**

Yoshifumi Mizuno, Research Center for Child Mental Development, University of Fukui, Fukui, Japan, email: mizunoy@u-fukui.ac.jp

**Online Resource 3: Other cognitive performances**

Cognitive data are shown in **Fig. 2a** of the main text and in **Online Resource 3 Fig**. In the main effect of group in the linear mixed-effect model, there were no significant differences between the NDD-A type and TD group for the TMT-A (*β* = -0.10 95% CI [-0.68, 0.48], *R*^2^ = 0.001, *t* = 0.34, *p*-FDR = 0.731, *p*-FWE > 0.999), TMT-B (*β* = 0.37, 95% CI [-0.20, 0.95], *R*^2^ = 0.02, *t* = 1.27, *p*-FDR = 0.308, *p*-FWE = 0.617), TMT-Δ (*β* = 0.48, 95% CI [-0.08, 1.06], *R*^2^ = 0.04, *t* = 1.65, *p*-FDR = 0.304, *p*-FWE = 0.609), stop signal task (stop signal reaction time, *β* = -0.17, 95% CI [-0.75, 0.40], *R*^2^ = 0.03, *t* = 0.60, *p*-FDR = 0.862, *p*-FWE > 0.999; direction errors on go trials, *β* = -0.003, 95% CI [-0.59, 0.58], *R*^2^ = 0.0005, *t* = 0.01, *p*-FDR = 0.991, *p*-FWE > 0.999; direction errors on stop trials, *β* = 0.16, 95% CI [-0.41, 0.75], *R*^2^ = 0.03, *t* = 0.56, *p*-FDR = 0.862, *p*-FWE > 0.999), and spatial working memory task (between errors, *β* = 0.40, 95% CI [-0.17, 0.99], *R*^2^ = 0.03, *t* = 1.36, *p*-FDR = 0.175, *p*-FWE = 0.351; within errors, *β* = 0.66, 95% CI [0.09, 1.23], *R*^2^ = 0.05, *t* = 2.30, *p*-FDR = 0.046, *p*-FWE = 0.093). Moreover, there were no significant differences between the NDD-B type and TD groups for the TMT-A (*β* = 0.25, 95% CI [-0.08, 0.59], *R*^2^ = 0.32, *t* = 1.47, *p*-FDR = 0.142, *p*-FWE = 0.285), stop signal reaction time (*β* = 0.16, 95% CI [-0.16, 0.49], *R*^2^ = 0.33, *t* = 0.96, *p*-FDR = 0.336, *p*-FWE = 0.672) and direction error on stop trials (*β* = 0.37, 95% CI [-0.003, 0.75], *R*^2^ = 0.15, *t* = 1.94, *p*-FDR = 0.082, *p*-FWE = 0.164) in the stop signal task, and spatial working memory task (between errors, *β* = 0.35, 95% CI [-0.007, 0.72], *R*^2^ = 0.15, *t* = 1.92, *p*-FDR = 0.113, *p*-FWE = 0.227; within errors, *β* = 0.03, 95% CI [-0.34, 0.41], *R*^2^ = 0.03, *t* = 0.17, *p*-FDR = 0.859, *p*-FWE > 0.999).

Online Resource 3 Fig. Cognitive results for each NDD subtype and the TD group. The NDD-A and NDD-B types did not show significant differences for the TMT-A, stop signal reaction time and direction errors on stop trials in the SST, and between errors and within errors in the SWMT. NDD, neurodevelopmental disorder; SST, stop signal task; SWMT, spatial working memory task; TD, typical development; TMT, trail making test.
