# Supplementary material for: Transdiagnostic monoamine-based subtyping for attention-deficit/hyperactivity disorder and autism spectrum disorder via unsupervised machine learning
Source: J Neural Transm (Vienna). 2026 Jul 27;133(8):1937–52. doi: 10.1007/s00702-026-03206-z (PMC13545128; doi:10.1007/s00702-026-03206-z)
Supplement: Supplementary file 4 [file 702_2026_3206_MOESM4_ESM.docx]

**Journal of Neural Transmission**

**Transdiagnostic monoamine-based subtyping for attention-deficit/hyperactivity disorder and autism spectrum disorder via unsupervised machine learning**

Masatoshi Yamashita^*^, Qiulu Shou, Sayo Hamatani, Hideo Matsuzaki, Masanori Fujieda, Yoshiyuki Hirano, Kuriko Kagitani-Shimono, Hidehiko Okazawa, and Yoshifumi Mizuno^#^

***Corresponding author**

Masatoshi Yamashita, Research Center for Child Mental Development, Hamamatsu University School of Medicine, Shizuoka, Japan, email: ymasa09@hama-med.ac.jp

**^#^Co-corresponding author**

Yoshifumi Mizuno, Research Center for Child Mental Development, University of Fukui, Fukui, Japan, email: mizunoy@u-fukui.ac.jp

**Online Resource 4 Table.** Brain areas with significantly smaller gray matter volume in the NDD-B type than in the TD group

|  | MNI coordinates | | |  |  |  |
| --- | --- | --- | --- | --- | --- | --- |
| Location | x | y | z | Cluster size | TFCE | *p*-FWE |
| Left central and inferior frontal opercular regions | -51 | 6 | 2 | 58972 | 418.77 | 0.001 |
| Right superior parietal lobule | 38 | -60 | 60 | 70 | 141.11 | 0.015 |
| Right supramarginal gyrus | 39 | -39 | 36 | 253 | 130.60 | 0.016 |

For the largest cluster (k = 58972), additional subpeaks were observed in the right inferior frontal opercular region, left precentral gyrus, left posterior orbital gyrus, right medial orbital gyrus, left frontal operculum, right triangular part of the inferior frontal gyrus, and bilateral subcallosal areas. FWE, family-wise error; MNI, Montreal Neurological Institute; NDD, neurodevelopmental disorder; TD, typical development; TFCE, threshold-free cluster enhancement.
